# High Glucose Variability Increases 30-Day Readmission Rates in Patients with Type 2 Diabetes Hospitalized in Department of Surgery

**DOI:** 10.1038/s41598-019-50751-7

**Published:** 2019-10-02

**Authors:** Ching Jung Hsieh

**Affiliations:** 1 Department of Internal Medicine, Pao Chien Hospital, Ping Tung, Taiwan, ROC; 2 Department of Nursing, College of Health and Nursing, Mei Ho University, Ping Tung, Taiwan

**Keywords:** Diabetes, Endocrinology

## Abstract

Glucose variability is common among hospitalized patients with type 2 diabetes mellitus (DM). I investigated to assess the variability of glucose in patients with type 2 DM accounts for in-hospital readmission rates in department of Surgery. I retrospectively analyzed 206 patients with type 2 DM, who was admitted to our hospital for surgical interventions and re-admitted within 30 days after discharge. I also enrolled 610 age, sex and diabetic duration matched patients with type 2 DM, as control. Outcomes measure included average and standard deviation (SD) of blood glucose during admission, glycated hemoglobin (HbA1c), lipid profile, renal function, length of stay (LOS). Patients who had re-admission within 30 days after discharge had higher SD of blood glucose levels than control (84.7 ± 53.5 mg/dL vs. 46.2 ± 42.8 mg/dL, *p* < 0.001) but not average of blood glucose levels. Comparing to control group, the study group also had higher HbA1c (8.4 ± 1.3% vs. 7.7 ± 1.1%, *p* = 0.015) and LOS (8.5 ± 2.5 days vs 7.0 ± 1.5 days, *p* = 0.020). The independent predictors of 30-day readmission rates were SD of blood glucose during admission and HbA1c (hazard ratio: 1.680, 1.493; *p* value < 0.001, 0.008, respectively). Decreasing glucose variability during admission for surgery is important for patients with type 2 DM to decreasing re-admission rates and LOS. HBA1c may also identify patients at higher risk of postoperative complications and possibility of re-admission.

## Introduction

Many published studies have shown that hyperglycemia correlates with mortality and morbidity in patients in hospital. In intensive care unit, highest mortality rates is also revealed in patients in the upper glucose quartile and having the highest every hour’s mean blood glucose change^1^. In critically or even in non-critically ill patients regardless with diabetes or not, well glycemic control could decrease hospital mortality, the length of stay (LOS), and 30-day readmission rate^2,3^. Comparing to primary service team in non-critical care units, inpatient diabetes with a specialized diabetes team consultation during the first day of admission could reduce admission cost, 30-day readmission rate apparently. The earlier consultation also significantly shorter hospital LOS and improves adherence to follow-up and transition of care^4^.

The hospitalized patients with diabetes mellitus (DM) have higher thirty-day readmission rates than all other hospitalized patients (14.4–22.7% vs. 8.5–13.5%)^5^. Hospital readmission is being used as a quality indicator and has been resulted in payment incentives in our health-care insurance policy. In the department of surgery of our hospital, the 30-day’s readmission rate is about 8.2% in all patients and 13.1% in patients with hyperglycemia noted during admission.

Glucose variability is the extent of blood glucose oscillations that occur within a specified period. From the results of continue blood glucose measurement, patients with DM with same mean blood glucose levels or glycated hemoglobin (HbA1c) may not have the same glucose profiles. Oscillation in blood glucose levels could lead to an overproduction of free radicals then inducing oxidative stress, especially in patients with type 2 DM^6–8^. In two of my previous studies, we found that reducing in that variability of blood glucose levels could increase the availability of antioxidants and decreased microvascular complications^9,10^. Therefore, steady and consistent blood glucose levels may decrease adverse outcomes during hospitalizations, independently of mean glucose levels.

The purpose of this study was to investigate the relationship between glycemic variability and hospitalization outcomes in patients with type 2 diabetes who were admitted to the Surgery wards of our hospital. In addition, as increased glycemic variability may also associate with an increase of LOS, so the in-hospital readmission rates may be increased.

## Materials and Methods

This is a single-center, hospital-based, retrospective chart review analysis. No investigational or interventional medication was provided. The variables of all patient were recorded in electronic medical charts.

### Subjects

I retrospectively followed the medical charts of 206 patients with type 2 DM (128 women and 78 men, age 56.4 ± 12.8 years), who was admitted to our hospital for surgical interventions and re-admitted within 30 days since January 2007 to December 2016. The surgical procedures underwent included 103 (50%) patients who received total knee replacement surgery, 43 (21%) patients who received total hip replacement surgery, 21 (10%) patients who received gall stone surgery, 21 (10%) patients who received renal stone surgery and 18 (9%) patients who received breast cancer surgery. All patients were confirmed to have type 2 DM based on 2017 American Diabetes Association diagnostic criteria. They remained hospitalized in the ward of surgical department for more than 4 days. During hospitalization, they all received a standard diabetic diet education according to the guideline of Taiwanese Association of Diabetes Educators (25 kcal/kg of ideal body weight, 55% of carbohydrate, 30% of fat, and 15% of protein) arranged by a certificated dietician. Medications were adjusted by a physician based on blood glucose measured by glucose meter 4 times/day (before breakfast, before lunch, before dinner and sleep time). All patients received blood glucose measured 4 times/days.

For standardization purposes, I also enrolled 610 age, sex and duration of diabetes matched patients (375 women and 235 men, age 57.1 ± 11.1 years) with type 2 DM, who received the same types of surgery at the same year as control. The surgical procedures underwent included 305 (50%) patients who received total knee replacement surgery, 128 (21%) patients who received total hip replacement surgery, 61 (10%) patients who received gall stone surgery, 61 (10%) patients who received renal stone surgery and 55 (9%) patients who received breast cancer surgery. They also received type 2 DM diagnosis based on 2017 American Diabetes Association diagnostic criteria and remained hospitalized in the ward of surgical department for more than 4 days. They had the same diabetic education, medication adjusting and blood glucose measuring as the study group.

I excluded any participant (including study and control group) who had a history or record of cardiogenic shock, unstable angina, old stroke, and myocardial infarction, renal function impairment (creatinine level >1.4 mg/dl), and liver cirrhosis history during enrollment period. I also excluded patient admitted with very long hospital stays (more than 60 days) to focus on acutely ill patients and patients with fewer than 5 glucose measurements during hospitalization.

### Outcome measures

Outcome measure from electronic medical record included average and standard deviation (SD) of blood glucose measurements during the hospital stay (blood glucose measured 4 times/days), glycated hemoglobin (HbA1c) within 1 month before and after admission, length of stay (LOS), medication for DM and co- morbidity. I also recorded diabetes microvascular complications (retinopathy, neuropathy and nephropathy). Patient’s history of hypertension and dyslipidemia was garnered from medical charts, as were new-onset coronary heart disease, stroke, and peripheral arterial occlusive disease. The diagnosis of coronary heart disease was based on electrocardiography findings or history of admission for percutaneous coronary intervention.

The following clinical and laboratory parameters were recorded during admission and within 1 month before and after admission from medical charts: body mass index (BMI), serum levels of total cholesterol (TC), low-density lipoprotein (LDL), high-density lipoprotein (HDL), triglyceride (TG), creatinine (Cr), and LOS.

This study was conducted according to the guidelines of the Declaration of Helsinki. The research protocol was approved by the Ethics Committee of the Pao Chien Hospital. The design of study method was similar to my previous study^11^.

### Assays

The concentrations of TC, LDL, HDL, TG and Cr (including urine Cr) were measured using an autoanalyzer (Hitachi 7250 Special; Hitachi, Tokyo, Japan). Urine albumin concentrations were determined by immunonephelometry (Dade-Behring, Marburg, Germany). The HbA1c level was measured by high-pressure liquid chromatography (Bio-Rad Laboratories, Inc, Richmond, CA, USA).

### Statistical analysis

The variables were summarized descriptively as mean ± SD; categorical variables are presented as number (%). Differences in clinical and biochemical characteristics (including age, gender distribution, body weight, LOS, SD of blood glucose, HbA1c, TC, LDL, HDL, TG, Cr, and UACR) between study and control group were tested using an unpaired *t*-test. Simple linear correlations were calculated by determining Pearson’s correlation coefficient r. Multiple regression models were used to investigate the influence of 30-day readmission rates. A probability value of <0.05 was considered significant. All statistical operations were performed using SPSS for Windows (Version 11.5; SPSS, Chicago, IL).

### Ethical approval

This study was approved by the Institutional Review Board of Pao Chien Medical Foundation on 2016/11/07. The IRB is organized and operates according to Good Clinical Practice and the applicable laws and regulations. This is a retrospective study. The need for informed consent was waived by the IRB.

## Results

### Study cohort

**Figure 1:**
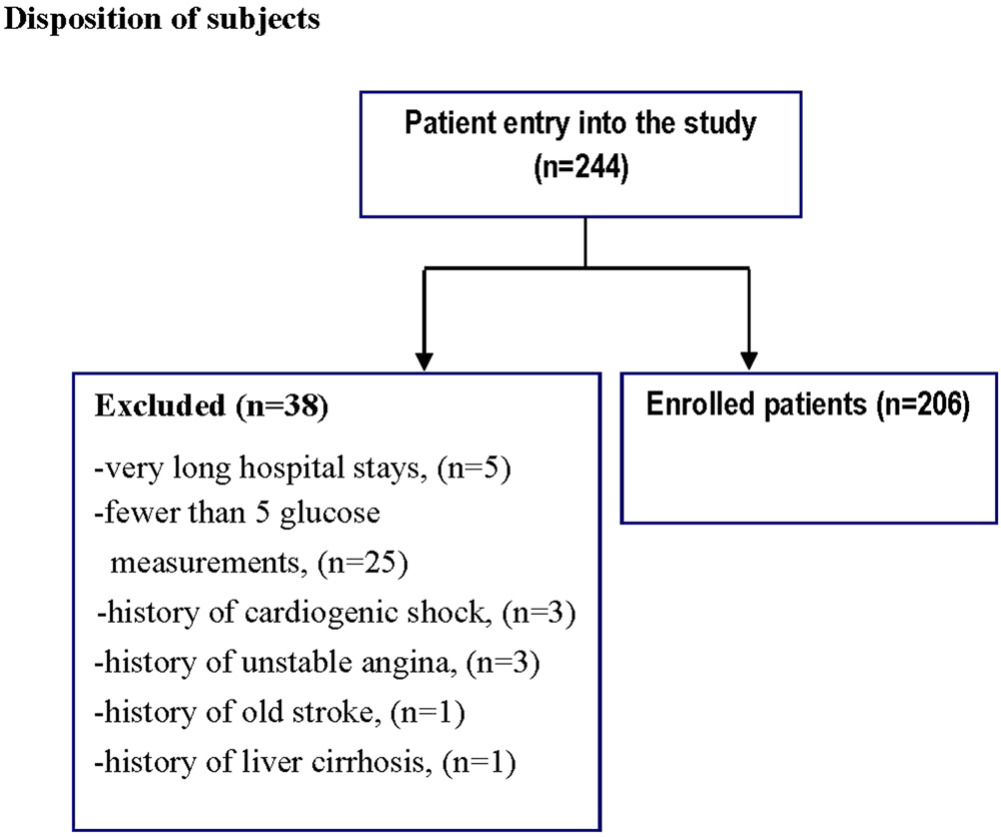
Disposition of subjects.

There are 244 patients with type 2 DM, who was admitted to our hospital for surgical interventions and re-admitted within 30 days since January 2007 to December 2016. After exclusion of participants who had a history or record of cardiogenic shock, unstable angina, old stroke, and myocardial infarction, renal function impairment (creatinine level >1.4 mg/dl), and liver cirrhosis history during enrollment period and who were admitted with very long hospital stays (more than 60 days) and patients with fewer than 5 glucose measurements during hospitalization. The final cohort included 206 (M: 78, F: 128; mean age: 56.4 ± 12.8 years) patients (Fig. 1). The all-cause 30-day readmission rate was 13.1% in patients with hyperglycemia. Wound infection or non-healing was the most common cause for readmission (71%). Severe dysglycemia accounted for 24% of 30-day readmission (14% hyperglycemia, 10%hypoglycemia), 2.5%of readmissions were coronary artery disease and 2.5% of unknown causes.

### Anthropometric and laboratory data

Patients who had re-admission within 30 days after discharge had higher SD of blood glucose levels than control (84.7 ± 53.5 mg/dL vs. 46.2 ± 42.8 mg/dL, *p* < 0.001) but not average of blood glucose levels (165.7 ± 27.5 mg/dL vs. 158.3 ± 27.2 mg/dL, *p* = 0.088). Comparing to control group, the study group also had higher HbA1c (8.4 ± 1.3% vs. 7.7 ± 1.1%, *p* = 0.015) and LOS (8.5 ± 2.5 days vs 7.0 ± 1.5 days, *p* = 0.020). There were no group differences in body weight, blood pressure, lipid profile, medication and co-morbidity (Table 1).

**Table 1:** Presents subjects’ demographic, clinical and biochemical characteristics.

Number	Re-admission within 30 days	Non-readmission within 30 days	*P-* value
	206 (M: 78, F: 128)	610 (M: 235, F: 375)	
Age (year)	56.4 ± 12.8	57.1 ± 11.1	0.55
Duration of DM (year)	10.6 ± 7.8	10.7 ± 6.6	0.86
Body weight (kg)	66.2 ± 12.5	67.9 ± 13.1	ns
Body height (cm)	161.8 ± 18.4	160.6 ± 18.1	ns
SBP (mmHg)	141.8 ± 24.8	139.8 ± 22.8	ns
DBP (mmHg)	78.9 ± 12.5	77.7 ± 11.7	ns
HbA1c (%)	8.4 ± 1.3	7.7 ± 1.1	0.02
Average of BG (mg/dL)	165.7 ± 57.5	158.3 ± 57.2	ns
SD of BG (mg/dL)	84.7 ± 53.5	46.2 ± 42.8	<0.001
Creatinine (mg/dL)	1.1 ± 0.6	1.0 ± 0.6	ns
TC	190.7 ± 36.8	189.8 ± 42.8	ns
HDL	46.9 ± 11.9	47.9 ± 11.3	ns
LDL	119.3 ± 33.2	117.5 ± 37.0	
TG	148.8 ± 89.5	150.5 ± 109.0	ns
Medication			
Diet control (%)	2	3	ns
OAD (%)	82	89	ns
Insulin (%)	20	18	ns
Complication			
retinopathy (%)	8	11	ns
nephropathy (%)	19	16	ns
neuropathy (%)	5	6	ns
Co-mobility			
Hypertension (%)	45	43	ns
Dyslipidemia (%)	34	36	ns
CAD (%)	11	9	ns
Stroke (%)	6	8	ns
PAOD (%)	4	5	ns
LOS	8.5 ± 2.5	7.0 ± 1.5	0.02

Abbreviations: SBP, systolic blood pressure; DBP, diastolic blood pressure; HbA1c, glycated hemoglobin; BG blood glucose; SD, standard deviation; TC, total cholesterol; HDL, high-density lipoprotein; LDL, low-density lipoprotein; TG, triglyceride; OAD, oral antidiabetic drug; CAD, coronary artery disease; PAOD, peripheral artery occlusion disease.

### Relationship between markers of diabetic control and other metabolic markers

**Figure 2:**
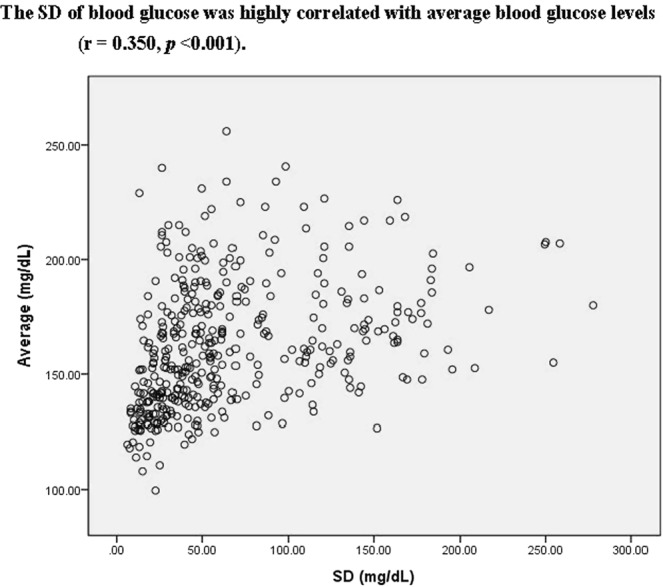
The SD of blood glucose was highly correlated with average blood glucose levels (r = 0.350, *p* < 0.001).

**Figure 3:**
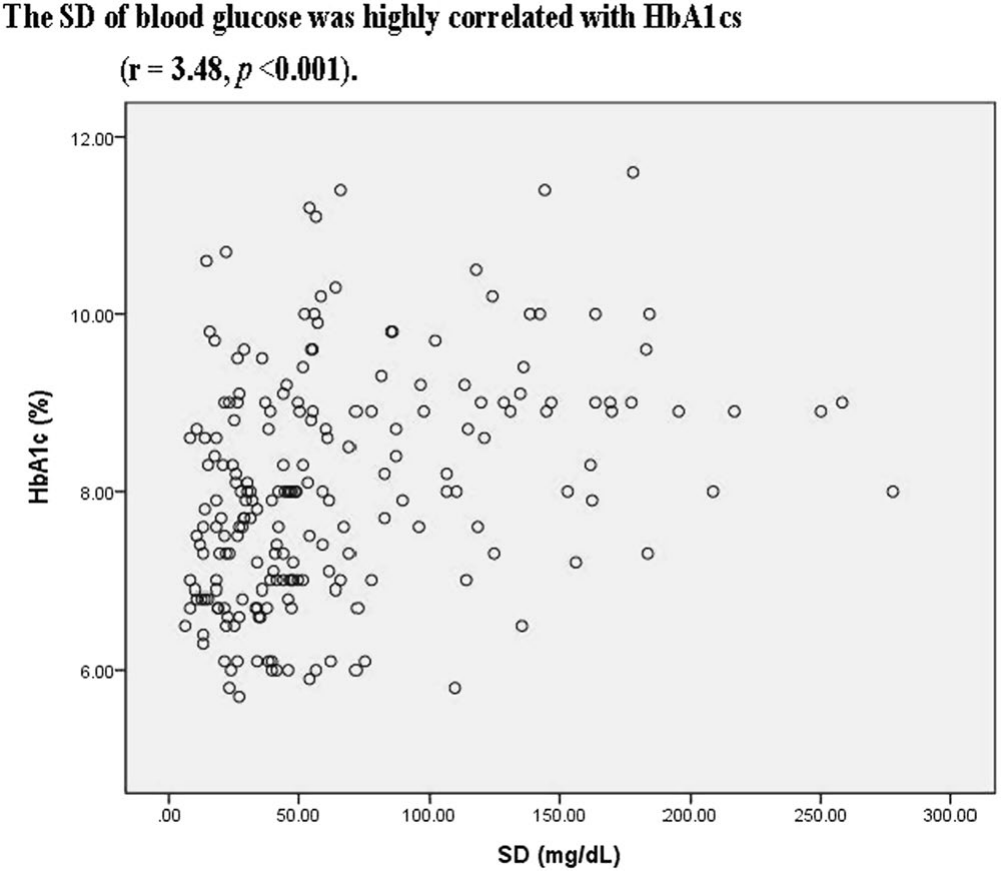
The SD of blood glucose was highly correlated with HbA1cs (r = 3.48, *p* < 0.001).

Table 2 shows the relationship between age, BMI, systolic blood pressure, diastolic blood pressure, HbA1c, average of blood glucose, SD of blood glucose levels, Cr, LDL, HDL TG and LOS for entire study group. SD of blood glucose was highly correlated with average blood glucose levels and HbA1cs (r = 0.35, 0.48 respectively, all *p* < 0.001, Figs 2 and 3). SD of blood glucose levels negatively correlated with BMI (r = −0.21, *p* = 0.002). The average blood glucose was only negatively correlated with BMI but not HbA1c (r = −0.17, *p* = 0.003; r = 0.06, *p* = 0.364, respectively). The LOS was also apparently correlated with SD of blood glucose levels, marginal correlated with HbA1c but not with average of blood glucose levels (r = 0.20, *p* = 0.003; r = 0.18, *p* = 0.031).

**Table 2:** Pearson’s correlation coefficients for markers of diabetic control and other metabolic markers.

	Age	BMI	SBP	DBP	HbA1c	ABG	SD	Cr	HDL	LDL	TG
BMI (kg/m^2^)	0.01										
SBP (mmHg)	0.18*	0.21*									
DBP (mmHg)	−0.07	0.22*	0.78*								
HbA1c (%)	−0.11	−0.00	−0.05	−0.03							
ABG (mg/dL)	−0.09	−0.17*	−0.00	−0.03	0.06						
SD (mg/dL)	−0.05	−0.21*	−0.07	−0.05	0.35*	0.35*					
Cr (mg/dL)	0.18*	0.06	0.16*	0.09	−0.02	0.01	0.00				
HDL (mg/dL)	0.02	−0.18*	0.04	0.04	−0.12	0.04	−0.08	−0.08			
LDL (mg/dL)	−0.03	0.05	0.15*	0.14	0.08	0.10	0.02	0.07	0.03		
TG (mg/dL)	−0.08	0.18*	0.06	0.02	0.12	0.10^#^	0.00	0.10^#^	−0.38*	0.10^#^	
LOS (days)	0.20*	0.08	0.05	−004.	0.18^#^	0.09	0.03	0.07	−0.08	0.11	0.03

Abbreviations: BMI, body mass index; SBP, systolic blood pressure; DBP, diastolic blood pressure; HbA1c, glycated hemoglobin; ABG average of blood glucose; SD, standard deviation; TC, total cholesterol; HDL, high-density lipoprotein; LDL, low-density lipoprotein; TG, triglyceride; LOS. Length of stay.

*p < 0.01; ^#^*p* < 0.05.

### Multivariate analysis

To assess the effects of different markers on 30-day readmission events, I performed the Mmultivariate Cox proportional-hazards models analyses. The markers included age, BMI, systolic blood pressure, diastolic blood pressure, HbA1c, average of blood glucose, SD of blood glucose levels, Cr, LDL, HDL, TG and LOS (Table 3). Patients with 30-day readmission had higher SD of blood glucose and HbA1cs than patients without 30-day readmission (hazard ratio: 1.680, 1.493; 95% confidence interval: 1.411~1.870, 1.214~1.683; *p* value < 0.001, 0.008, respectively). Prolong stay in the hospital was also revealed in the group with 30-days readmission (hazard ratio: 1.300; 95% confidence interval: 1.204~1.483; *p* value = 0.020).

**Table 3:** Multivariate analysis of risk factors for readmission (Cox’s model).

Variables	HR	95% CI	*P* value
Age	0.977	0.888~1.008	0.644
BMI	1.020	0.9892~1.110	0.757
SD of BG	1.680	1.411~1.870	<0.001
HbA1c	1.493	1.214~1.683	0.008
SBP	1.127	1.001~1.380	0.053
DBP	1.050	1.001~1.187	0.090
Cr	1,077	0.790~1.387	0.591
HDL	1.009	0.990~1.022	0.667
LDL	1.010	0997~1.003	0.354
TG	1.100	0.999~1.201	0.085
LOS	1.300	1.204~1.483	0.020

The dependent variable was 30-days readmission. The independent variables were age, body mass index, average of BG, SD of of BG, HbA1c levels, SBP, DBP, Cr, LDL, HDL, TG and LOS. Abbreviations: BMI: body mass index; HbA1c: glycated hemoglobin; SD: standard deviation of glycated hemoglobin; Cr: creatinine; HDL: high-density lipoprotein; LDL: low-density lipoprotein; TG: triglyceride; LOS: length of stay. HR: hazard ratio; 95% CI: 95% confidence.

Multiple linear regression analyses were performed to which markers of diabetic control predicted length of days before re-admission (Table 4). The markers also included age, BMI, systolic blood pressure, diastolic blood pressure, HbA1c, average of blood glucose, SD of blood glucose levels, Cr, LDL, HDL, TG and LOS. Using generalized estimating equations, multivariate regression analysis revealed that SD of blood glucose (*p* < 0.001) and HbA1c (*P* = 0.009) were both independent predictors of days before re-admission.

**Table 4:** Multivariate linear regression analysis (Total r^2^: 0.812).

Variables	Beta coefficient	T	*P* value
SD (mg/dL)	0.284	3.727	<0.001
HbA1c (%)	0.187	2.650	0.009
LOS (days)	0.143	2.005	0.046
SBP (mmHg)	−0.100	−1.384	0.168
Average of BG (mg/dL)	0.012	0.164	0.870

Notes: Dependent variable: days before re-admission. r^2^ for entire model = 0.812.

The dependent variable was days before re-admission. The independent variables were age, BMI (body mass index), SBP (systolic blood pressure), DBP (diastolic blood pressure), HbA1c, average of BG (blood glucose), SD (standard deviation) of blood glucose levels, Creatinine, HDL (high-density lipoprotein), LDL (low-density lipoprotein), TG (triglyceride) and LOS (length of stay).

## Discussion

In patients of DM, the risk of worse clinical outcomes during admission, including wound infections, renal function impairment and longer LOS increase significantly^12^. Preoperative HbA1c may be an apparent risk factor of post-operative complications, especially higher HbA1c related to infection were also revealed in surgical intervention of gynaecological cancer^13^. In this study, I found the correlation between 30-days readmission rate and poor blood glucose control. The patients who had higher HbA1c before, during or after surgical intervention, got more chance to be re-admitted within 30 days. The patients with type 2 diabetes had longer hospitalization also higher rate of-30-days’ re-admission rate. In the previous study, well glycemic control to <180 mg/dL during admission can reduce hospital mortality, 30-day readmission rate and average LOS even in non-critically ill patients^3^.

Oscillation in blood glucose levels could lead to an overproduction of free radicals then inducing oxidative stress, especially in patients with type 2 DM^6–8^. In my previous study, every five minutes’ acute fluctuation and chronic blood glucose variability (SD of HbA1c levels) significantly correlated with increasing oxidative stress markers: urine 8-isoprostaglandin F2α, serum thiobarbituric acid-reactive substance, and serum 8-hydroxydeoxyguanosine. Glycemic fluctuation can also increase the serum levels of chronic inflammatory marker (high-sensitivity C-reactive protein) in my previous study^9^. In my previous another study, the more variability of blood glucose levels, the less antioxidants were measured and more microvascular complications revealed^10,14^. Therefore, fluctuation of blood glucose levels could increase LOS and frequency of re-admission via influencing wound healing and infection. In a prospective observational study in cardiac surgery patients reveals that glycemic variability was a significant predictor of length of stay in intensive care unit and rise in creatinine after surgery. In this investigation, patients with 30-day readmission had higher SD of blood glucose, which was also a strong independent predictor of length of days before re-admission. Glycemic variability is therefore a new dimension in postoperative glycemic management in cardiac surgery patients^15^. In another study of coronary artery bypass surgery, patients transferred from intensive care unit with increased glycemic variability combined with elevated preoperative HbA1c could predict adverse outcomes^16^. My study’s result also revealed instead of glycemic variability, HbA1c before, during or after admission also to be an important predictor of 30-day readmission rates.

Obesity significantly increased the risk of a postoperative wound complication and infection^17^. The obese habitus and physiology significantly affects patients undergoing otologic and neurologic surgeries^18^. The patient with lower BMI may have less complication of surgery, short LOS and less 30-day re-admission rate. However, conflict results were revealed. Sergesketter *et al*. concluded obesity may not have a significant impact on surgical outcomes and 30 days’ readmission rate after cranial surgery^19^. Even in two studies of spine surgery, one revealed preoperative obesity is an independent risk factor for readmission within 30 days of discharge but another showed equivalent clinical outcomes found among obese and non-obese patients treated for lumbar spine stenosis^20,21^. Wang *et al*. conducted a cross-sectional study, which reveals lower BMI associated with increased glycemic variability, characterized by elevated post-prandial blood glucose excursion in newly diagnosed Chinese patients of type 2 diabetes^22^. In my study, we also found that SD and average of blood glucose levels were all negatively correlated with BMI. Although BMI was not a good predictor of readmission rate in multivariate regression analysis, the relationship between BMI, glycemic variability and postoperative wound complication may need further investigation.

There are several limitations in this study. First, risk factors for readmission may include lower socioeconomic status, racial/ethnic minority, comorbidity burden, and emergent or urgent admission. Because this is a retrospective study, I can’t analyze socioeconomic status and admission status. However, in the real-world clinical setting, there were no group differences in body weight, blood pressure, lipid profile, medication and co-morbidity. All patients with type 2 diabetes received inpatient education, specialty care, discharge instructions, coordination of care, and post-discharge support. Second, measuring post-prandial blood glucose were not performed in the study and control groups because of the limitation of insurance payment in our country. Therefore, glycemic excursion is less reliable than 7-point blood glucose profiles. Third, beta cell function plays an important role to determinate blood glucose variability, especially in different stages of diabetes. However, measuring beta cell function can’t be achieved in all enrolled patient in this retrospective study. For standardization purposes and comparing to study group, I enrolled age, sex and duration of diabetes matched patients with type 2 DM as control.

In conclusion, glucose fluctuation will increase the LOS and 30-day re-admission rate in patients with type 2 diabetes mellitus. Decreasing glucose variability during hospitalization for surgery is important. Well blood glucose control to target HbA1c also plays an important role to reducing the risk of postoperative complications and possibility of re-admission.

## Data Availability

All relevant data are available through Figshare.com with the following DOI: https://springernature.figshare.com/s/ef479fdaf63c50db70be.
